# A Treatise on the Incubus or Night-Mare, Disturbed Sleep, Terrific Dreams, and Nocturnal Visions; with the Means of Removing These Distressing Complaints

**Published:** 1816-09

**Authors:** 


### PART II.

### COMPREHENSIVE ANALYTICAL REVIEW OF MEDICAL LITERATURE.

"Tros, tyriusve, nobis nullo discrimine agetur."

*A Treatise on the Incubus or Night-Mare, disturbed Sleep, terrific Dreams, and nocturnal Visions; with the Means of removing these distressing Complaints.* By JOHN WALLER, Esq. Surgeon R.N. 8vo. pp. 115. London, 1816.

THE frightful hag has so often chased "tired Nature's sweet restorer" from our couch, and rode triumphant o'er our struggling frames, that we anxiously perused the little work which we now usher into notice. We were gratified at finding a very great coincidence of opinion between our author and ourselves; and, taught by long and painful experience, we can vouch for the faithful delineations and judicious observations of Mr. Waller.

*Refreshing sleep* is not only such a criterion of health, but such a solace of our woes, and such a zest to our waking enjoyments, that an investigation of any disturbance thereto, is not, we conceive, beneath the dignity of the greatest medical philosopher. We shall therefore dedicate a larger proportion of our work to the subject of this little volume, than its size or importance might otherwise seem to demand, confident that utility and amusement will result from its perusal.

Mr. Waller has been a sufferer from this complaint for nearly twenty years, and consequently writes from personal experience, as well as much reading, reflection, and inquiry on the subject. He considers, and we think justly, that disturbed sleep, frightful dreams, and terrific visions, are all so many grades of night-mare, which is the climax of the disorder. He believes that the vulgar opinion of "*bad dreams*" being the forerunners of diseases is not chimerical, though they will frequently occur, also, where the disturbance of the system is comparatively trifling, as in slight derangements of the digestive organs, and biliary system in particular. On this account, children are very subject to the milder degrees of Incubus: but to adults, the attacks of night-mare are still more distressing, as being indicative of more serious derangement of the system; and, upon the whole, are by no means to be ranked amongst the lesser calamities to which our nature is liable. Many there are, who are wont to rise from their beds in the morning more wearied and exhausted than when they retired to sleep; which last indeed, to them, is an object of terror rather than comfort! Such a life cannot long be borne with impunity; and serious, if not fatal diseases must be the consequence.

Incubus was observed and described at a very early period. This, as well as the Greek term *Ephialtes*, is expressive of the weight and oppression felt, and conveys the idea of some *living being* having taken its position on the breast, inspiring terror, impeding respiration, and paralysing all the voluntary muscles. It is not wonderful to find such an extraordinary affection ascribed to dæmoniacal agency; an idea which has prevailed in all ages and countries. Its real nature has not till this hour been satisfactorily explained, and modern physicians pay little attention to its investigation: hence the victims of Incubus are left without a remedy, and almost without a hope!

We can bear witness with Mr. Waller, that many of the modern opinions respecting night-mare are absolutely erroneous; such as, that it only happens to persons lying upon their backs, and who have eaten large suppers, &c. On these premises, the causes have been ascribed to mechanical pressure on the lungs from an extended stomach; and abstinence from food in the evening, with a lateral position in bed, is all that is supposed necessary to prevent its attacks. Those, however, who are subject to this complaint in any great degree, know the fallacy of these doctrines.

The hypothesis of Dr. Darwin, that Incubus is occasioned by too sound sleep, and that no real difficulty of breathing exists, is contradicted by the experience and feelings of every victim to the malady.

Hippocrates must have known, though he does not describe, Incubus. Some allusion to the disease is made by Galen: "Verum *ephialtes* longo tempore perdurans in epilepsiam convertitur;" an opinion embraced by Mr. Waller himself. Cælius Aurelianus relates a remarkable circumstance, on the authority of Silimachus, namely, that this affection was once *epidemic* at Rome, and that many died of it. Mr. Waller accounts for this passage by another in Sylvius de Leboe, who has recorded the history of an epidemic disease at Leyden, in 1669, in which Incubus was a very common symptom.

It is a certain fact, however, that the higher degrees of Incubus are by no means devoid of danger. Mr. Waller knew one instance in which a paroxysm of it certainly proved fatal, and he has heard of several others.

"A young man of sober habits, about 30 years of age, had been subject to severe attacks of night-mare all his life. During the paroxysms, he frequently struggled violently, and vociferated loudly. Being at Norwich, on some business which detained him there several weeks, he, one night, retired to bed in apparent good health. In the night, or towards morning, he was heard, by some of the family in the house where he lodged, to vociferate and groan as he had been accustomed to do during the paroxysms of night-mare; but as he was, after no great length of time, perfectly quiet, no person went to his assistance. In the morning he was found dead, having thrown himself, by his exertions and struggles, out of bed, with his feet still entangled among the bed-clothes."

We agree with Mr. Waller, that the above unfortunate man died, in all human probability, of Incubus! In the severer forms of this disease, the shock of falling out of bed is not near so effectual in rousing the person afflicted, as the voice or touch of another person. Mr. W. thinks, that Incubus is frequently the forerunner of, and occasionally terminates in epilepsy.

Incubus has been elegantly described by poets, and correctly by physicians.

Ac velut in somnis, oculos ubi languida pressit

Nocte quies, nequicquam avidos extendere cursus

Velle videmur, et in mediis conatibus ægri

Succidimus; non lingua valet, non corpore notæ

Sufficiunt vires, nec vox, nec verba sequuntur.

Æneid xii. v. 909.

The celebrated modern Caledonian bard has also drawn a picture of Incubus:

In broken dreams the image rose
Of varied perils, pains, and woes;
His steed now flounders in the brake,
Now sinks his barge upon the lake;
Now leader of a broken host,
His standards fall—his honour's lost.
Then—from my couch may heavenly might
Chase that worst phantom of the Night!
*Lady of the Lake.*

*Description of Incubus.* Whatever may be the evidence of the senses to the contrary, the disease always attacks during sleep. The first approach of the fiend is, if the sleep be profound, in the shape of a disagreeable dream. The patient imagines himself exposed to some danger, or pursued by an enemy which he cannot avoid He frequently feels as though his limbs were tied or deprived of motion; at other times he fancies himself confined at the bottom of a cavern or vault, and in danger of suffocation. This is often the whole of the sensation which the disease produces, when it goes off either by an oblivious sleep, or a pleasant dream. Here Incubus is not fully formed; the predisposition to it only is evinced.

"When the paroxysm does actually take place, the uneasiness of the patient in his dream rapidly increases, till it ends in a kind of consciousness that he is in bed and asleep; but he feels to be oppressed with some weight, which confines him on his back and prevents his breathing, which is now become extremely laborious, so that the lungs cannot be fully inflated by any effort he can make. The sensation is now the most painful that can be conceived: the person becomes every instant more awake and conscious of his situation; he makes violent efforts to move his limbs, especially his arms, with the view of throwing off the incumbent weight; but not a muscle will obey the impulse of the will: he groans aloud, if he has strength to do it, while every effort he makes seems to exhaust the little remaining vigour. The difficulty of breathing goes on increasing, so that every breath he draws seems to be almost the last that he is likely to draw; the heart generally moves with increased velocity, sometimes is affected with palpitation; the countenance appears ghastly, and the eyes are half open. The patient, if left to himself, lies in this state, generally about a minute or two, when he recovers all at once the power of volition: upon which he either jumps out of bed, or instantly changes his position, so as to wake himself thoroughly. If this be not done, the paroxysm is very apt to recur again immediately, as the propensity to sleep is almost irresistible, and, if yielded to, another paroxysm of night-mare is, for the most part, inevitable." 24.

This is an excellent delineation of the paroxysm of Incubus. Where the disease is established, some confusion of head, singing in the ears, and spectra before the eyes, will remain for a time after being roused. In proportion to the establishment of ephialtes, there will also be acceleration of pulse and palpitation of the heart. To these our author adds priapismus, a sense of we ght at the stomach, and an unpleasant taste in the mouth.

Mr. Waller believes, that when the paroxysm goes off, as frequently happens, without the patient waking, strange hallucinations are produced, which give origin to reputed visions and supernatural visitation, even among people of great intellectual cultivation. The degree of consciousness during a paroxysm of night-mare is so much greater than ever happens in a dream, that the person who has had a vision of this kind cannot easily bring himself to acknowledge the deceit, unless he wakes out of the paroxysm, and finds some incongruity in respect to time or place, which proves the transaction an illusion. Of the various deceptions of this kind related by Mr. Waller, we shall here select one.

"Mr. B—— (at this moment a student in London) was once living in lodgings in the vicinity of St. Thomas's Hospital; and happening to wake in the middle of the night, as he imagined, he heard the sounds of footsteps approach his door, which was quickly opened, and he saw distinctly a man enter the room, whom he described as having on a blue coat with white buttons; the moon was shining into the room, and he could see every object distinctly: the man approached the side of the bed, when Mr. B—— drew himself under the bed-clothes; in this situation, he heard distinctly the ticking of his watch under the pillow, where he had always taken the precaution to secure it. In a short time, he felt the hand of the man rummaging the pillow, as if with the design of seizing the watch; upon which Mr. B—— drew the watch gently into the bed, and concealed it there: he still, however, felt distinctly the man's hand under the pillow, and was now in the greatest alarm imaginable, not only for his watch but for his personal safety, and began to complain aloud of pain in the bowels, accusing the supper he had eaten as being the cause of the disturbance, with the idea, that by this stratagem he might succeed in getting up and going out of the room, without exciting any suspicion in the man, who was still (as he supposed) standing by the bed-side, as to the true cause of his getting up. He at length ventured to get out on the opposite side of the bed, and hastened towards the door: the man followed him, and he says he felt distinctly the impression of his hand upon one shoulder, just as he was escaping out of the door. He ran instantly into the bed-room of the man who kept the house, and gave an alarm. This person immediately arose and called in the watchman: the house was searched from top to bottom very strictly, but no person of any description could be found; the doors and windows were all secure, nor was there a possibility of any one getting in or out of the house unobserved. Mr. B——, however, could not be satisfied on this score; the evidence of his own senses, which had never before deceived him, appeared to him to be superior to all other evidence whatsoever. He quitted his lodgings the next day, and retained pertinaciously the opinion, that what he had seen was real, until more than a year afterwards, when, beingat sea, he was again visited by this extraordinary affection, and was equally certain of the reality of his vision: but in this case, he had the opportunity of proving, in the most satisfactory manner, that it was a delusion." 39.

We have no doubt, that in this way many well-meaning persons have deceived themselves and others with the belief of having seen spectres, heard voices, &c. in the dead of the night, and very possibly that such convictions have, under certain circumstances, produced the event foreboded or dreaded.

The visions accompanying paroxysms of Incubus are often of the most terrific kind, as every person subject to this distressing complaint will allow.

" *Forrestus incubo affectus putabat pectus suum comprimi a cane nigro, unde respirare non potuit, utut fœmina videret esse somnium fallax, uti de se refert.*"

Many of the Greek and Roman writers attributed the whole phænomena of Ephialtes to the agency of Demons. St. Augustine considered it as such, and speaks of it as a thing very common in those days.

" *Dæmones scilicet, qui mulieribus se commiscent et ab incubando* INCUBI *dicunter, sicuti, qui viris, et patiuntur muliebria* SUCCUBI." —De Civitate Dei.

To a modification of Incubus, Mr. Waller attributes that indescribable terror which some persons feel in their sleep, and which frequently obliges them to vociferate aloud, and generally to start with violence, or even jump out of bed, either with or without a terrific dream. This terror is often connected with some object not at all terrific in itself, as a cat, dog, or sometimes a little child. These will be contemplated a while in a dream without dread; but, all at once, will become objects of horror and alarm, and that without changing appearance or attitude. The terror produced in this way sometimes continues for several minutes after we are awake, and is almost always accompanied with a sensation of shivering, such as we feel at the rehearsal of a tale of horror.

*Of the persons most subject to night-mare.*—Incubus will sometimes occur in the healthiest person, when any indigestible food happens to lie in his stomach, or the upper portions of the alimentary canal, during sleep. But a peculiar habit of body is necessary to render a person *subject* to it. Many feel it from youth. These are generally of a contemplative disposition, and of that particular temperament which disposes to hypochondriasis and nervous diseases. Sedentary employments, confinement within doors, literary studies, &c. also predispose to the attacks of Incubus. Those, too, who are accustomed to coarse and unwholesome food, however exposed to the open air, are obnoxious to this disease; hence its great prevalence among sailors. Hypochondriacs and pregnant women are also its victims; but the male sex are much more frequently so than the female. It is not very common in advanced life, except where corpulency, tendency to lethargy, or asthma exists.

*The proximate cause* of Incubus has given rise to various speculations among physicians; not one, probably, of which is right. A very generally adopted opinion, however, that this affection is produced by mechanical obstruction to the blood's circulation from particular position of the body, deserves notice. It is a certain fact, for which we also can vouch, that no position of the body is a security from night-mare among the predisposed. Neither is a full stomach to be accused as the cause, nor an empty one to be expected as the antidote of this disorder. Mr. Waller, as well as ourselves, took every possible precaution to avoid these and other reputed causes of Incubus, but in vain—indeed, often with a manifest aggravation of the complaint. The perpendicular position of the body, we have experienced to be the worst in which a paroxysm of night-mare can be borne; the difficulty of respiration, and other symptoms being greatly increased, besides a new symptom being added—a perpetual dread of falling, which appears inevitable, and prevents the patient from struggling, as in the horizontal posture. Next to this is the position of sleeping with the body bent forwards, and the head reclining with the face downwards on a table. No difference exists between recumbence on the right or left side. The almost universal opinion, that Incubus attacks persons *only* while on their backs, must have some reason; and the following seems to be it.

" It appears to be one of the symptoms almost inseparable from the disease, that the patient should *appear to himself* to be kept down upon the back by some external force. This sensation I have almost always felt, even when I have had the evidence of others, as well as my own conviction, when awake, that I was in reality lying on the side. There is also another sensation which is very apt to deceive the patient;—that is, on the paroxysm going off, and the moment of his recovering the power of volition, a great confusion of ideas always takes place, and a person to whom the night-mare is not very familiar, generally imagines that he has recovered himself by some effort of his own, frequently by turning from his back to his side, sometimes by sitting upright in bed. These things are always extremely fallacious; there is no trusting to the senses during a paroxysm of Incubus."

We can amply confirm the above statement by our own experience.

Mr. Waller acknowledges that he cannot satisfactorily account for the proximate cause, or *modus agendi* of this disease; and therefore, we shall not quote his attempts to elucidate it; but the following passage will throw some light on the remote cause and the means of prevention, as well as treatment.

"It appears evident, from the mode of treatment to which this disease gives way, that the primary cause, in whatever manner it may act, has its seat in the digestive organs; nor can any difference, or even error, in explaining its *modus agendi*, in any manner invalidate this doctrine. The ancient physicians seem tolerably well to agree on this point, and consider night-mare as a species of epilepsy, arising from a superabundance of *acid humour* in the primæ viæ, and their treatment was entirely directed to the evacuation and correction of that humour. If they were mistaken in their pathological ideas of this affection, they were, at all events, successful in their treatment; and it was usually their practice to reason from the cure to the cause of a disease, a safe mode of reasoning in medicine. Experience has taught them that a long train of diseases originated from this cause, and a little observation will suffice to convince any man, unprejudiced by medical theories, that night-mare originates from no other cause than a defect in the digestive process, whereby the food, which should be converted into good chyle, is transformed into a half-digested mass of acid matter, which is productive of heart-burn, acid eructations, flatulence, and intolerable gripings, with the whole train of dyspeptic and hypochondriacal symptoms. There are many stomachs which convert every thing they receive instantly into an acid, and such will be generally found to be the case with persons subjected to habitual night-mare, or frightful dreams, and disturbed sleep. Such stomachs too are frequently distended with some acid gas, and I have often found the paroxysm of night-mare to be the consequence of this distension alone, and to be instantly removed by any thing which would dispel the gas, such as a glass of peppermint cordial, or gin, or any carminative medicine. A medical gentleman in Norfolk, who laboured under an inveterate disease of the digestive organs for some years, which in the end proved fatal, was dreadfully tormented by the most pertinacious paroxysms of night-mare, which threatened suffocation, had recourse to a solution of ammonia in warm water, which he always drank when the paroxysms of night-mare began to disturb him; for when they did begin, they always continued to torment him every time he fell asleep. The success of this plan far exceeded his expectations. The immediate effect of the medicine, was to send up a great quantity of wind from the stomach, which was succeeded by a profuse perspiration, and tranquil undisturbed sleep."

Mr. W. always found, in his own case, that night-mare was accompanied with distension of the stomach and bowels by flatus, constipation, and acid eructations; and his enquiries led to the conclusion that others were similarly affected. In short, he became convinced, that his complaint was altogether dependent on the state of dyspepsia, under which he laboured; for, on going into the country, and his digestive organs becoming more regular in their functions, the Incubus in a great measure disappeared.

Observing the success of the ammonia in the case abovementioned, he had recourse to it himself; but it did not agree with his stomach: other alkalies were tried, but with no better success. At length he fixed on carbonate of soda, dissolved in ale or porter, which he found a delicious beverage. Mr. W. generally used about a drachm in the day of the soda; but whenever he found a sense of oppression about the præcordia, which indicated the predisposition to night mare, he had immediate recourse to a large dose of the medicine, and always obtained relief.

"This salt not only corrects the acidity in the *primæ viæ*, but likewise brings away by stool a quantity of viscid slimy matter, so acrid as to burn and excoriate the parts as it passes out. As I constantly persisted in the use of this alkaline salt, and got rid of this trash from the body, my appetite, which had long been lost and depraved, returned, and the digestive organs performed their functions again with ease and success. But still the propensity to acidifying remains, and a cautious attention to diet, and to the evacuations, is always necessary. By perseverance in a plan founded on these principles, I found my enemy at length subdued, and brought under a degree of control which I had never hoped to obtain, and this circumstance has induced me to give to the world my opinions and advice. By a close attention to the latter, I do not doubt but they will succeed in driving from their couch this fiend of night—this enemy of repose." 103.

A small work on this subject, by Bond, published some fifty years ago, recommends blood-letting. Mr. W. thinks that this remedy would be inadmissible in the majority of persons habitually affected with this complaint. We much doubt this, however, and we know at this moment a medical gentleman who was completely freed from the attacks of Incubus by a large bleeding.

There are certain kinds of food which will constantly produce this disease in the predisposed. Hildeshiem says, "*Qui scire cupit quid sit Incubus? Is ante somnum comedat castaneas, et superbibat vinum fæculentum!*"

This we believe. A tart or fruit pie that is at all acid, is sure to give us the company of this frightful fiend during the night. The articles most likely, in this country, to bring on night-mare, are, Mr. W. thinks, cucumbers, nuts, apples, and all flatulent kinds of food. The following form is that which Mr. Waller recommends as a night draught for the prevention of Incubus.

℞. Potass. carbonat. gr. x ; tinct. card. C. ʒiij ; syrupi simplicis, ʒi ; aquæ menthæ pip. ℥j ; M. fiat haustus. H. S. S.—Or the following: ℞. Ammon. pp. gr. x ; tinct. capsici, ʒj ; syr. croci, ʒj ; aq. cinnamon, ʒx ; M. fiat haustus.—In the event of these medicines failing to keep the bowels open, a dose of neutral salts, or magnesia, rhubarb, and carbonate of potash in mint water, should be taken the succeeding morning.

Intemperance of every kind is hurtful, but particularly the drinking of bad wine. Of eatables, fat and greasy meats, most vegetables, fruit and pastry, are to be avoided, or used very sparingly. The same may be said of salted meat, which is very improper for dyspeptic persons, though they are generally fond of it. Moderate exercise is beneficial; sedentary employments, and particularly intense study, with late hours, are highly prejudicial. But going to bed before the usual hour frequently brings on a paroxysm; the same takes place from indulging in sleep too late in the morning.

Persons affected with Incubus should always have some one sleep near them, so as to be immediately awoke by their groans or struggles. The sooner a person is roused from a paroxysm the better, since, in a high degree, it differs little from a fit of epilepsy. Where medicine is not at hand, a glass of common gin, or other cordial, will frequently dispel flatulence and prevent the paroxysm of Incubus.

But temporary relief is not merely to be kept in view by the patient; the permanent amendment of his constitution, and the prevention, if possible, of the formation of acid and unhealthy secretions in the alimentary canal, should be the paramount object. This will require a long perseverance in the plan here laid down, in order to eradicate the disease.

"Indeed," says Mr. Waller, "I would recommend them never to drink any malt liquor without a portion of the carbonate of soda, or some other alkaline salt in it, and to pay the greatest attention to regularity and choice of diet. One of the draughts before mentioned, or any thing of the same nature which may be found more agreeable, should be taken, whenever the dyspeptic symptoms are at all urgent, and repeated as often as occasion may require. Costiveness should be obviated, and where there is much languor or debility, with loss of appetite, I would recommend the *pil. ferri c. myrrha*, with decoct. cinchon. infusion of gentian, quassia, or other agreeable bitter." 114.

We have thus exhibited a very full analysis of this interesting little volume, and assigned it a much greater space than its size, or even the subject might seem to demand, convinced that sufficient attention has not, of late, been paid to a harassing and distressing infirmity of our nature. We can say, from personal experience, that we have tried Mr. Waller's prescription, and have found it effectual in preventing Incubus. We were in the habit of taking the pil. hyd. twice or thrice a week, with soda water at intervals, but we have found the carbonate of soda more effectual, and it certainly forms a very pleasant beverage in ale or porter.

Mr. Waller was not unknown to the medical world before the present publication, and we confidently anticipate still more important communications from his pen. The little work which we have analysed contains intrinsic proof of intellectual strength, and intellectual cultivation.

